# RTK with the Assistance of an IMU-Based Pedestrian Navigation Algorithm for Smartphones

**DOI:** 10.3390/s19143228

**Published:** 2019-07-22

**Authors:** Zun Niu, Ping Nie, Lin Tao, Junren Sun, Bocheng Zhu

**Affiliations:** School of Electronics Engineering and Computer Science, Peking University, Beijing 100871, China

**Keywords:** GNSS, android smartphones, RTK, AHRS, ZUPT, MEMS-IMU

## Abstract

Real-time kinematic (RTK) technique is widely used in modern society because of its high accuracy and real-time positioning. The appearance of Android P and the application of BCM47755 chipset make it possible to use single-frequency RTK and dual-frequency RTK on smartphones. The Xiaomi Mi 8 is the first dual-frequency Global Navigation Satellite System (GNSS) smartphone equipped with BCM47755 chipset. However, the performance of RTK in urban areas is much poorer compared with its performance under the open sky because the satellite signals can be blocked by the buildings and trees. RTK can’t provide the positioning results in some specific areas such as the urban canyons and the crossings under an overpass. This paper combines RTK with an IMU-based pedestrian navigation algorithm. We utilize attitude and heading reference system (AHRS) algorithm and zero velocity update (ZUPT) algorithm based on micro electro mechanical systems (MEMS) inertial measurement unit (IMU) in smartphones to assist RTK for the sake of improving positioning performance in urban areas. Some tests are carried out to verify the performance of RTK on the Xiaomi Mi 8 and we respectively assess the performances of RTK with and without the assistance of an IMU-based pedestrian navigation algorithm in urban areas. Results on actual tests show RTK with the assistance of an IMU-based pedestrian navigation algorithm is more robust and adaptable to complex environments than that without it.

## 1. Introduction

Over the past two decades, GNSS has been evolving rapidly [1]. The U.S. government has promoted the Global Positioning System (GPS) modernization program, adding the second civil signal on L2 frequency (L2C) and the third civilian signal on L5 frequency (L5). As of 24 April 2019, there were a total of 31 operational satellites in the GPS constellation, not including the decommissioned, on-orbit spares [2]. Europe is developing the Galileo system to provide global services. Up to 12 February 2019, there were a total of 22 satellites in operation [3]. Galileo navigation signals are transmitted in the following four frequency bands: E1, E5a, E5b, and E6 bands. The frequency of E1 is equal to that of L1 of GPS (1575.42 MHz), while the frequency of E5a is equal to that of L5 of GPS (1176.45 MHz) [4].

RTK was first proposed as a differential positioning technology that processes both the pseudorange and carrier phase measurements of GPS to provide cm-level accuracy in real time [5]. RTK involves at least one stationary receiver and at least one movable receiver. The stationary receiver, which is called the base station, plays the role of the reference to calculate the position of the movable receiver, which is called the rover [6]. Solving the ambiguity of the carrier phase is one of the most important procedures of RTK. The revolution of GNSS improves the performance of RTK thanks to the increasing number of visible satellites and civilian signals. Utilizing other constellations in addition to GPS improves spatial distribution and reduces problems caused by obstructions, which improves the accuracy and availability of RTK, as shown for GPS/Galileo [7], GPS/GLONASS [8], GPS/BDS [9], and GPS/BDS/Galileo/QZSS [10]. RTK also benefits from the dual frequency, as the accuracy and ambiguity resolution performance are improved [11,12]. Compared with geodetic-grade dual-frequency and multi-frequency receivers, consumer-grade receivers are more widely used due to their low costs. However, low-cost commercial receivers often use inexpensive hardware, especially antennas with poor performance, which leads to the lower carrier-to-noise ratio (CNR), worse resistance to multipath, and poorer ambiguity resolution performance [13]. However, smartphones are often equipped with low-cost receivers. The antennas on smartphones are often linearly polarized (LP) [14], while circular polarization (CP) is generally adopted in satellites to avoid Faraday rotation problems [15]. As a result, the smartphone’s antenna has a loss in the power of signals received, which leads to lower CNR.

Before 2016, Raw GNSS measurements collected by a smartphone were not available to users. Most users only had access to the position computed by the GNSS chipsets. A software-defined receiver called GRIDwas adopted to generate measurements from signals collected by smartphone antennas. The test showed that poor multipath suppression of these antennas did harm to the integer ambiguity resolution [16]. Conditions had changed as Google introduced the Android 7.0 operating system in 2016, indicating that users could get raw GNSS measurements including pseudorange, Doppler, and carrier phase through some APIs. Raw measurements collected by a Samsung Galaxy S7 with the BCM4774 GNSS chipset were analyzed in November 2016. Results confirmed that the quality of carrier-phase measurements could potentially allow for a centimeter-level positioning. However, not only the poor quality of the antenna, but also the duty cycling prevent smartphones from being used as practical RTK units [17]. Duty cycling is a technique to prolong battery life. The duty cycling switches on the navigation chip for a fixed period before switching it off. This fixed period is called the burst period. The burst period can last for 200 milliseconds in one second. What happens in the remaining 800 milliseconds is not known [18,19]. The duty cycling prevents the smartphone from tracking the carrier phase continuously. Carrier phase collected during several minutes after a cold start of a Huawei P10 was used to provide precise position. Results showed the smartphone could reach decimeter accuracy in static conditions and sub-meter when used in urban vehicle scenarios [18]. The Nexus 9 tablet with BCM4752 was one of few devices that had the duty cycling feature disabled before May 2018. Tests using the carrier phase collected by the Nexus 9 tablet for precise positioning proved that not integer phase ambiguities, but float solutions could be estimated. It also proved that precise positioning was feasible with a moderate level of multipath [20].

There were only chipsets supporting the single frequency of each constellation such as BCM4774 and BCM4752 mentioned above. These chipsets differed in supporting different kinds of constellations. A new revolution happened in May 2018 as the first dual-frequency GNSS smartphone produced by Xiaomi was launched. The Xiaomi Mi 8 could collect dual-frequency (L1/E1 + L5/E5a) raw GNSS measurements with the BCM47755 chipset. Meanwhile, Google introduced the Android P operating system that could disable duty cycling with the developer option “Force full GNSS measurements” [21]. These two changes made precise positioning on smartphones much more promising. Frank van Diggelen, the Principal Engineer at Google, pointed out that some phones disabled the duty cycling automatically when users requested raw measurements [22]. The NSL’s FLAMINGO (Nottingham Scientific Limited’s fulfilling enhanced location accuracy in the mass-market through Initial Galileo Services) Team found that the carrier phase collected by the Xiaomi Mi 8 was not affected by the duty cycling [23,24]. Static differential positioning using the carrier phase of GPS and Galileo (L1/E1 + L5/E5a) on a very short baseline provided cm-level precision in the horizontal component and decimeter-level in the vertical component [19]. Observations and positioning performance were analyzed in different multipath conditions, indicating that the Xiaomi MI 8 was very promising to provide sufficiently accurate positioning results in urban areas [25]. With dual-frequency raw measurements, the Xiaomi Mi 8 could use the combination of L1 bands and L5 bands to eliminate most ionosphere errors, which helped obtain more accurate positioning results than smartphones like the Samsung S8 utilizing single-frequency measurements [26]. The carrier phase measured by the Xiaomi Mi 8 could also be used for precise point positioning (PPP) [27].

Users of smartphones are often pedestrians in urban areas. They are more likely to walk through an urban canyon, which is a narrow street with very tall buildings on both sides. They also often walk under overpasses. These overpasses and obscurations can cause the receiver to lose track of the signals, therefore leading to the loss of the carrier phase for many seconds [28]. During that time, RTK cannot provide valid positioning results. The outage of signals hinders all kinds of GNSS positioning methodologies. One common way to improve this is to integrate GNSS with the inertial navigation system (INS). INS is based on an IMU, which consists of gyroscopes and accelerometers, enabling computing velocity, attitude, and position. A hybrid IMU containing magnetometers can also be called a magnetic, angular rate, and gravity (MARG) sensor, which gives more accurate orientation [29]. Assisting GNSS with INS can reduce acquisition time and improve immunity to noise and interference [30]. When it comes to RTK, ambiguity resolution and cycle slip detection benefit from INS aiding [31,32]. Single-frequency multi-constellation RTK tightly coupled with INS performs better than dual-frequency RTK without INS in urban areas [33]. We can couple RTK with INS to assist ambiguity resolution and resist noise, but no position output can be gained frequently in GNSS-challenged environments and GNSS-denied environments. The positioning results are provided by the INS during the GNSS outage [34]. However, INS based on an MEMS-IMU that suffers from large drift cannot provide satisfactory positioning results for a long period of time [35].

Navigation algorithms based on inertial MEMS sensors are divided into two types in general: pedestrian dead reckoning (PDR) algorithms and INS-based algorithms [36]. PDR estimates the position by counting steps and estimating stride length and heading. Many algorithms, such as peak detection and zero-crossing counting, can be used to detect and count steps [37]. After successfully detecting a new step, PDR uses a kind of step length model to estimate the moving distance of a pedestrian [38]. AHRS algorithms can be used to improve the accuracy of estimating the heading angle, which improves the PDR’s performance [39,40]. INS uses strapdown mechanical equations to estimate position, velocity, and attitude. INS-based algorithms utilize some pseudo-measurements to correct the estimate of position, velocity, biases of sensors, and attitude [41]. For example, a foot-mounted INS has explicit stance phases. The velocity should be zero during the stance phases so the ZUPT algorithm can be used to utilize the zero-velocity to reduce the long-term drifts of IMU [42]. PDR algorithms and INS-based algorithms can be coupled for a more accurate estimate of heading and position [36].

In this paper, we collect observation measurements through a Xiaomi Mi 8. We compare the performance of single-frequency RTK with that of dual-frequency RTK based on the Xiaomi Mi 8 in both static mode and dynamic mode. We also compare the performance of RTK based on the Xiaomi Mi 8 with that based on a dual-frequency NovAtel receiver equipped with an OEM617-CDS-R0G-550 card. Here, we utilize RTKLIB [43] to take the measurements. We also provide a method to assist RTK with an IMU-based pedestrian navigation algorithm for smartphones. Considering GNSS outage and MEMS-IMU integrated into our smartphone, we adopt the Madgwick algorithm, which is one kind of AHRS algorithm to provide the initial attitude of our smartphone and utilize the ZUPT algorithm to aid INS to provide positioning results during GNSS outage.

The rest of the paper is organized as follows: In Section 2, the RTK algorithm, the Madgwick algorithm, the ZUPT algorithm, and the procedure of collecting data are introduced in detail. The performance comparison among RTK using the Xiaomi Mi 8, using the NovAtel receiver, and RTK with the assistance of an IMU-based pedestrian navigation algorithm are described in Section 3. Conclusions and future work are summarized in Section 4.

## 2. Materials and Methods

### 2.1. Implementation of RTK

We used RTKLIB to analyze the single-frequency and dual-frequency RTK performance based on the Xiaomi Mi 8. In this section, we introduce the theory of RTKLIB [43,44].

#### 2.1.1. EKF Formulation

RTKLIB is based on the extended Kalman filter (EKF). The EKF is a nonlinear version of the Kalman filter (KF), which can help solve nonlinear state estimation. A discrete nonlinear system at epoch $t_{k}$ can be described with two formulas:

(1) $${\hat{x}}_{k}=f\left(x_{k-1},u_{k},w_{k}\right)$$

(2) $$y_{k}=h\left(x_{k},v_{k}\right)$$

Here, ${\hat{x}}_{k}$ and $y_{k}$ are the estimated state vector and measurement vector at epoch time $t_{k}$, respectively. $w_{k}$ and $v_{k}$ are the process and observation noises, which are both assumed to be multivariate Gaussian noises with zero mean and covariance $Q_{k}$ and $R_{k}$, respectively. $u_{k}$ is defined as the control vector. We can utilize the process function *f* to compute the predicted state at epoch $t_{k-1}$ from the state at epoch $t_{k}$ and utilize measurement function *h* to compute the predicted measurement at epoch $t_{k}$ from the predicted state at epoch $t_{k}$. We define two Jacobian matrices $F_{k-1}^{k}$ and $H_{k}$ as the matrix of partial derivatives of function *f* and function *h*, respectively:

(3) $$F_{k-1}^{k}=\frac{\partial f}{\partial x}{|}_{{\hat{x}}_{k-1}}$$

(4) $$H_{k}=\frac{\partial h}{\partial x}{|}_{{\hat{x}}_{k}^{-}}$$

The procedure of EKF can be divided into two parts at each epoch: predicting and updating.

Predicting aims to provide predicted state estimate ${\hat{x}}_{k}^{-}$ and covariance estimate $P_{k}^{-}$ as follows:

(5) $${\hat{x}}_{k}^{-}=f\left({\hat{x}}_{k-1},u_{k},0\right)$$

(6) $${\hat{P}}_{k}^{-}=F_{k-1}^{k}{\hat{P}}_{k-1}{F_{k-1}^{k}}^{T}+Q_{k}$$

Updating aims to provide updated state estimate ${\hat{x}}_{k}$ and updated covariance estimate $P_{k}$ as follows:

(7) $$K_{k}={\hat{P}}_{k}^{-}{H_{k}}^{T}{\left(H_{k}{\hat{P}}_{k}^{-}{H_{k}}^{T}+R_{k}\right)}^{-1}$$

(8) $${\hat{x}}_{k}={\hat{x}}_{k}^{-}+K_{k}\left(y_{k}-h\left({\hat{x}}_{k}^{-},0\right)\right)$$

(9) $${\hat{P}}_{k}=\left(I-K_{k}H_{k}\right){\hat{P}}_{k}^{-}$$

#### 2.1.2. Theory of RTK

RTK is a differential positioning methodology that utilizes the double-differencing pseudorange and carrier phase. We present the principle of triple-frequency RTK for the sake of generality. We can use single-frequency RTK or dual-frequency RTK in practical applications. We define state vector ***x*** and measurement vector ***y*** as follows:

(10) $$x={\left({r_{r}}^{T},{v_{r}}^{T},{B_{1}}^{T},{B_{2}}^{T},{B_{5}}^{T}\right)}^{T}$$

(11) $$y={\left({\Phi_{1}}^{T},{\Phi_{2}}^{T},{\Phi_{5}}^{T},{P_{1}}^{T},{P_{2}}^{T},{P_{5}}^{T}\right)}^{T}$$

where $r_{r}$ and $v_{r}$ are the position and velocity of the rover, respectively; $B_{i}$ is the $L_{i}$ single-differencing carrier-phase biases. $\Phi_{i}$ and $P_{i}$ are the double-differencing phase-range and pseudorange measurements on the $L_{i}$ band, respectively. When it comes to the predicting procedure, we can define $F_{k-1}^{k}$ as Equation (12), considering basic kinematics theory and the consistency of the carrier biases if there is no loss of lock:

(12) $$F_{k-1}^{k}=\left[\begin{array}{ccc} I_{3\times 3} & I_{3\times 3}\Delta t & O_{3\times (3m-3)}\\ O_{3\times 3} & I_{3\times 3} & O_{3\times (3m-3)}\\ O_{(3m-3)\times 3} & O_{(3m-3)\times 3} & I_{\left(3m-3)\times (3m-3\right)} \end{array}\right]$$

When it comes to the updating procedure, we can define the measurement function h(x) as Equation (13), assuming there are *m* satellites in view:

(13) $$h(x)={\left({h_{\Phi ,1}}^{T},{h_{\Phi ,2}}^{T},{h_{\Phi ,5}}^{T},{h_{P,1}}^{T},{h_{P,2}}^{T},{h_{P,5}}^{T}\right)}^{T}$$

where:

(14) $$h_{\Phi ,i}=\left[\begin{array}{c} \rho_{rb}^{12}+\lambda_{i}\left(B_{rb}^{1}-B_{rb}^{2}\right)\\ \rho_{rb}^{13}+\lambda_{i}\left(B_{rb}^{1}-B_{rb}^{3}\right)\\ \vdots \\ \rho_{rb}^{1m}+\lambda_{i}\left(B_{rb}^{1}-B_{rb}^{m}\right) \end{array}\right]$$

(15) $$h_{P,i}=\left[\begin{array}{c} \rho_{rb}^{12}\\ \rho_{rb}^{13}\\ \vdots \\ \rho_{rb}^{1m} \end{array}\right]$$

In the two equations, $\lambda_{i}$ is the wavelength of the $L_{i}$ bands. $\rho_{rb}^{1k}$ is the double-differencing pseudorange of the 1st satellite and the kth satellite.

We define D as the single-differencing matrix:

(16) $$D=\left[\begin{array}{ccccc} 1 & -1 & 0 & \cdots & 0\\ 1 & 0 & -1 & \cdots & 0\\ \vdots & \vdots & \vdots & \ddots & \vdots \\ 1 & 0 & 0 & \cdots & -1 \end{array}\right]$$

We utilize $e_{r}^{k}$ to stand for the LOS vector from the receiver antenna to the kth satellite in ECEF. We can define E as:

(17) $$E=\left[\begin{array}{c} e_{r}^{1}\\ e_{r}^{2}\\ \vdots \\ e_{r}^{m} \end{array}\right]$$

Finally, we can write $H_{k}$ as:

(18) $$H_{k}=\left[\begin{array}{ccccc} -DE & O_{(m-1)\times 3} & \lambda_{1}D & O_{(m-1)\times m} & O_{(m-1)\times m}\\ -DE & O_{(m-1)\times 3} & O_{(m-1)\times m} & \lambda_{2}D & O_{(m-1)\times m}\\ -DE & O_{(m-1)\times 3} & O_{(m-1)\times m} & O_{(m-1)\times m} & \lambda_{5}D\\ -DE & O_{(m-1)\times 3} & O_{(m-1)\times m} & O_{(m-1)\times m} & O_{(m-1)\times m}\\ -DE & O_{(m-1)\times 3} & O_{(m-1)\times m} & O_{(m-1)\times m} & O_{(m-1)\times m}\\ -DE & O_{(m-1)\times 3} & O_{(m-1)\times m} & O_{(m-1)\times m} & O_{(m-1)\times m} \end{array}\right]$$

### 2.2. Introduction of the Madgwick Algorithm

The Madgwick algorithm can help provide attitude estimation with both IMU and MARG sensors. This algorithm introduced by Madgwick at the 2011 IEEE International Conference on Rehabilitation Robotics is one kind of AHRS algorithm [29,45]. The Madgwick algorithm employs a quaternion to describe the attitude.

#### 2.2.1. Introduction of the Quaternion

**Figure 1:**
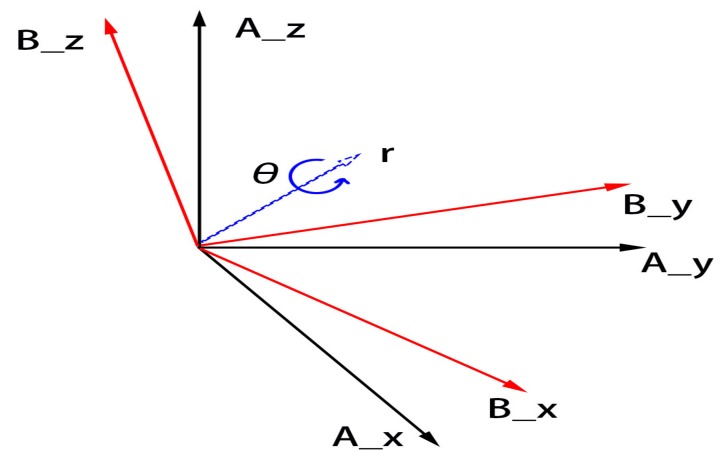
Visualization of frame A and frame B. Frame A rotates an angle θ around the r axis to become frame B.

We used a four-dimensional complex number called the quaternion to stand for the attitude of a rigid body relative to a coordinate frame in the three-dimensional space. For example, ${}_{B}^{A}\hat{q}$ reflects the attitude of frame B relative to frame A (Figure 1). We can compute the quaternion with the angle θ between frame B and frame A:

(19) $${}_{B}^{A}\hat{q}=\left[\begin{array}{c} q_{0}\\ q_{1}\\ q_{2}\\ q_{3} \end{array}\right]=\left[\begin{array}{c} cos(\frac{\theta }{2})\\ -r_{x}sin\left(\frac{\theta }{2}\right)\\ -r_{y}sin\left(\frac{\theta }{2}\right)\\ -r_{z}sin\left(\frac{\theta }{2}\right) \end{array}\right]$$

(20) $$r=\left[\begin{array}{c} r_{x}\\ r_{y}\\ r_{z} \end{array}\right]$$

Here, r stands for the orientation of the angle. We use ${}_{B}^{A}\hat{q^{*}}$ to denote the conjugate of ${}_{B}^{A}\hat{q}$, which represents the attitude of frame A relative to frame B. We can define ${}_{B}^{A}\hat{q^{*}}$ as:

(21) $${}_{B}^{A}\hat{q^{*}}{=}_{A}^{B}\hat{q}=\left[\begin{array}{c} q_{0}\\ -q_{1}\\ -q_{2}\\ -q_{3} \end{array}\right]$$

We can define ${}^{S}\omega$ as a quaternion that is formed with the components of the $\omega_{nb}^{b}$, assuming $\omega_{nb}^{b}$ is the angular rate:

(22) $$\omega_{nb}^{b}=\left[\begin{array}{c} \omega_{x}\\ \omega_{y}\\ \omega_{z} \end{array}\right]$$

(23) $${}^{S}\omega =\left[\begin{array}{c} 0\\ \omega_{x}\\ \omega_{y}\\ \omega_{z} \end{array}\right]$$

Then, we can write the quaternion differential equation as:

(24) $${}_{E}^{S}{\dot{q}}_{\omega ,t}=\frac{1}{2}{{}_{E}^{S}\hat{q}}_{est,t-1}⊗{}^{S}\omega_{t}$$

Here, ${}^{S}\omega_{t}$ is the angular rate measured at time *t*; ${}_{E}^{S}\hat{q}$ is the estimate of the attitude quaternion of the Earth frame relative to the sensor frame at time t−1; and ${}_{E}^{S}{\dot{q}}_{\omega ,t}$ is the quaternion derivative, which reflects the change rate of the quaternion. ⊗ is defined to denote quaternion multiplication. Then, we can compute the estimate of the attitude quaternion at time *t*, assuming Δt is the sample interval:

(25) $${}_{E}^{S}q_{\omega ,t}={{}_{E}^{S}\hat{q}}_{est,t-1}+{}_{E}^{S}{\dot{q}}_{\omega ,t}\Delta t$$

This quaternion is calculated with the measurements of gyroscopes.

#### 2.2.2. Theory of the Madgwick Algorithm

We can calculate the quaternion with the measurements of gyroscopes, as mentioned above. We can also calculate the quaternion with the measurements of the accelerometers and magnetometers. Suppose we had a predefined reference vector ${}^{E}\hat{d}$ in the field of the Earth frame. We can compute the reflect of the vector in the sensor frame using the quaternion:

(26) $${}^{S}\hat{d}={}_{E}^{S}{\hat{q}}^{*}{⊗}^{E}\hat{d}{⊗}_{E}^{S}\hat{q}$$

We can also get the value of vector ${}^{S}\hat{s}$ in the measured field of the sensor frame. We can define an objective function to represent the difference between ${}^{S}\hat{d}$ and ${}^{S}\hat{s}$ as follows:

(27) $$f{(}_{E}^{S}\hat{q}{,}^{E}\hat{d}{,}^{S}\hat{s}){=}^{S}\hat{d}{-}^{S}\hat{s}={}_{E}^{S}{\hat{q}}^{*}{⊗}^{E}\hat{d}{⊗}_{E}^{S}\hat{q}{-}^{S}\hat{s}$$

The most accurate quaternion should go with the minimum of the objective function. Therefore, this is an optimization problem that can be solved using gradient descent, as Equation (28) shows:

(28) $${}_{E}^{S}q_{k+1}={{}_{E}^{S}\hat{q}}_{k}-\mu \frac{\nabla f{(}_{E}^{S}{\hat{q}}_{k}{,}^{E}\hat{d}{,}^{S}\hat{s})}{\left|\left|\nabla f{(}_{E}^{S}{\hat{q}}_{k}{,}^{E}\hat{d}{,}^{S}\hat{s})\right|\right|}$$

In Equation (28) μ, stands for the variable step-size.

The objective function is formed with two sub-functions solving the measurements of accelerometers and magnetometers, respectively. When it comes to the measurements of accelerometers, we substitute ${}^{E}\hat{g}$ and normalized accelerometers measurements ${}^{E}\hat{a}$ for ${}^{S}\hat{d}$ and ${}^{S}\hat{s}$, respectively, as shown in Equations (29) and (30). Then, we can get the first sub-function, as shown in Equation (31).

(29) $${}^{E}\hat{g}=\left[\begin{array}{c} 0\\ 0\\ 0\\ 1 \end{array}\right]$$

(30) $${}^{S}\hat{a}=\left[\begin{array}{c} 0\\ a_{x}\\ a_{y}\\ a_{z} \end{array}\right]$$

(31) $$f_{g}{(}_{E}^{S}\hat{q}{,}^{E}\hat{g}{,}^{S}\hat{a})=\left[\begin{array}{c} 2\left(q_{1}q_{3}-q_{0}q_{2}\right)-a_{x}\\ 2\left(q_{0}q_{1}+q_{2}q_{3}\right)-a_{y}\\ 1-2\left(q_{1}^{2}+q_{2}^{2}\right)-a_{z} \end{array}\right]$$

When it comes to the measurements of magnetometers, we substitute the Earth’s magnetic field ${}^{E}\hat{b}$ and normalized magnetometer measurements ${}^{E}\hat{a}$ for ${}^{S}\hat{d}$ and ${}^{S}\hat{s}$, respectively, as shown in Equations (32) and (33). Then, we can get the second sub-function, as shown in Equation (34).

(32) $${}^{E}\hat{b}=\left[\begin{array}{c} 0\\ b_{x}\\ 0\\ b_{z} \end{array}\right]$$

(33) $${}^{S}\hat{m}=\left[\begin{array}{c} 0\\ m_{x}\\ m_{y}\\ m_{z} \end{array}\right]$$

(34) $$f_{b}{(}_{E}^{S}\hat{q}{,}^{E}\hat{b}{,}^{S}\hat{m})=\left[\begin{array}{c} 2b_{x}\left(0.5-q_{2}^{2}-q_{3}^{2}\right)+2b_{z}\left(q_{1}q_{3}-q_{0}q_{2}\right)-m_{x}\\ 2b_{x}\left(q_{1}q_{2}-q_{0}q_{3}\right)+2b_{z}\left(q_{0}q_{1}+q_{2}q_{3}\right)-m_{y}\\ 2b_{x}\left(q_{0}q_{2}+q_{1}q_{3}\right)+2b_{z}\left(0.5-q_{1}^{2}-q_{2}^{2}\right)-m_{z} \end{array}\right]$$

Then, we can form the objective function with two sub-functions as follows:

(35) $$f_{g,b}{(}_{E}^{S}\hat{q}{,}^{E}\hat{a}{,}^{E}\hat{b}{,}^{S}\hat{m})=\left[\begin{array}{c} f_{g}{(}_{E}^{S}\hat{q}{,}^{E}\hat{g}{,}^{S}\hat{a})\\ f_{b}{(}_{E}^{S}\hat{q}{,}^{E}\hat{b}{,}^{S}\hat{m}) \end{array}\right]$$

Then, we can calculate the quaternion using the gradient descent algorithm as Equation (36) shows:

(36) $${}_{E}^{S}q_{\nabla ,t}={}_{E}^{S}{\hat{q}}_{est,t-1}-\mu_{t}\frac{\nabla f_{g,b}{(}_{E}^{S}\hat{q}{,}^{E}\hat{a}{,}^{E}\hat{b}{,}^{S}\hat{m})}{\left|\left|\nabla f_{g,b}{(}_{E}^{S}\hat{q}{,}^{E}\hat{a}{,}^{E}\hat{b}{,}^{S}\hat{m})\right|\right|}$$

Here, we have two quaternions. One of them is calculated using the angular rate, while the other one is practically calculated utilizing the measurements of the accelerometers and magnetometers. The proper fusion of the two separate quaternions can be more accurate. Different weights can be applied to each quaternion to get the fusion as Equation (37) shows:

(37) $${}_{E}^{S}q_{est,t}=\gamma_{t}{}_{E}^{S}q_{\nabla ,t}+\left(1-\gamma_{t}\right){}_{E}^{S}q_{\omega ,t},0\leq \gamma_{t}\leq 1$$

Proper $\gamma_{t}$, $\mu_{t}$, and some deformation can help us get Equations (38) and (39) to calculate the fused quaternion.

(38) $${}_{E}^{S}q_{est,t}={}_{E}^{S}{\hat{q}}_{est,t-1}+{}_{E}^{S}{\dot{q}}_{est,t}\Delta t$$

(39) $${}_{E}^{S}{\dot{q}}_{est,t}={}_{E}^{S}{\dot{q}}_{\omega ,t}-\beta \frac{\nabla f_{g,b}{(}_{E}^{S}\hat{q}{,}^{E}\hat{a}{,}^{E}\hat{b}{,}^{S}\hat{m})}{\left|\left|\nabla f_{g,b}{(}_{E}^{S}\hat{q}{,}^{E}\hat{a}{,}^{E}\hat{b}{,}^{S}\hat{m})\right|\right|}$$

β in Equation (39) stands for the divergence rate of ${}_{E}^{S}q_{\omega }$ because of the drift.

### 2.3. Introduction of ZUPT Aiding INS

ZUPT is widely used in INS-based pedestrian navigation. ZUPT is based on a foot-mounted IMU, which means we must fix the IMU on our foot. The ZUPT aiding INS can be divided into two procedures: zero-velocity detection and updating state with virtual measurements [46,47].

#### 2.3.1. Zero-Velocity Detection

The walking process of pedestrians can be modeled as a repeating sequence of heel strike, stance, push off, and swing. The velocity of our foot should be zero during the stance phase when the foot is bearing the weight of the whole body. The zero-velocity can be utilized as a “virtual measurement” to correct the IMU’s attitude, velocity, and position if we can detect the occurrence of the zero-velocity. While there are many approaches to help detect zero-velocity, we chose the generalized likelihood ratio test (GLRT) to derive the detector [48]. The IMU is considered stationary at epoch *k* if Equation (40) is satisfied.

(40) $$\frac{1}{N}\sum_{i\in W_{k}}(\frac{1}{\sigma_{f}}-\left|\left|f_{i}-g\frac{\bar{f_{k}}}{\left|\left|\bar{f_{i}}\right|\right|}\right|\right|+\frac{1}{\sigma_{\omega }}{\left|\left|\omega_{i}\right|\right|}^{2})<\gamma$$

In Equation (40), $\bar{f_{k}}$ is the mean of accelerometer measurements over the time window $W_{k}$ of length *N* samples adjacent to epoch *k*. $\sigma_{f}$ and $\sigma_{\omega }$ reflect the error of accelerometer and gyroscope, respectively. ${\bar{f}}_{i}$ and $\omega_{i}$ are the samples at epoch *i* in the window.

#### 2.3.2. Updating the State with Virtual Measurements

We used KF to predict and update states. The predicting can be described as Equation (41), considering we chose the position, velocity, and the attitude quaternion as state variables.

(41) $$\left[\begin{array}{c} x_{k}\\ v_{k}\\ q_{k} \end{array}\right]=\left[\begin{array}{c} x_{k-1}+v_{k-1}dt_{k}\\ v_{k-1}+\left(q_{k-1}f_{k}q_{k-1}^{-1}-g\right)dt_{k}\\ \Omega \left(\omega_{k}dt_{k}\right)q_{k-1} \end{array}\right]$$

In Equation (41), $x_{k}$ is the position, $v_{k}$ is the velocity, and $q_{k}$ is the attitude quaternion. $q_{k}^{-1}$ is the conjugate of $q_{k}$. $f_{k}$ and $\omega_{k}$ are measurements in three dimensions. Ω(·) is the quaternion updating matrix, which is relative to the angular rate and angle. We can obtain the virtual residual $Z_{k}$ when the zero-velocity occurs as Equation (42) shows.

(42) $$Z_{k}=\left[\begin{array}{c} 0-v_{x,k}\\ 0-v_{y,k}\\ 0-v_{z,k} \end{array}\right]$$

Then, we can calculate the Kalman filter feedback assuming $K_{k}$ is the Kalman gain to correct the states as Equation (43) shows.

(43) $${dx}_{k}=K_{k}Z_{k}$$

### 2.4. RTK with the Assistance of an IMU-Based Pedestrian Navigation Algorithm

We walked through an urban canyon. RTK could not provide reliable and continuous positioning results in the urban canyon because of GNSS outage. The assistance of an IMU-based pedestrian navigation algorithm could be helpful. We tied the Xiaomi Mi 8 to our foot, and the Xiaomi Mi 8 collected the measurements of the accelerometer, the gyroscopes, and the magnetometers. By applying the Madgwick algorithm, we could estimate and record the Xiaomi Mi 8’s attitude with these measurements. We counted the interval between two positioning results output by RTK. If the intervals between a positioning result and its last output and also its next output both exceeded the threshold, this positioning result would be ignored. The last valid positioning result before the GNSS outage was regarded as the initial position of the ZUPT aiding INS. We synchronized the outputs of RTK and the Madgwick algorithm so that we could provide the initial attitude, especially the yaw, for the ZUPT aiding INS. The outputs of the ZUPT aiding INS were regarded as positioning results during GNSS outage in the urban canyon. Once we passed through the city canyon, the ZUPT aiding INS stopped outputting positioning results, and RTK continued providing positioning results.

### 2.5. Data Collection

We used the Xiaomi Mi 8 to collect the GNSS raw measurements and the measurements of IMU.

#### 2.5.1. GNSS Raw Measurements’ Collection

Here, we used an app called Geo++ RINEX Logger, which could produce a file of measurements in RINEX format [49]. There are other similar apps such as RINEX ON [50]. RINEX ON has the advantage of providing not only measurements, but also ephemeris. However, not all kinds of smartphones provide the users with ephemeris. Google also released the GnssLogger app to log the data related to GNSS measurements [51]. GnssLogger did not directly provide pseudorange or carrier phase. Users could only calculate the pseudorange and carrier phase with the data provided by GnssLogger.

Many other smartphones are equipped with BCM47755 and Android P. We can refer to the Android Developers Documentation to choose one kind of phone to develop some applications or test the RTK and PPP performance [52].

**Figure 2:**
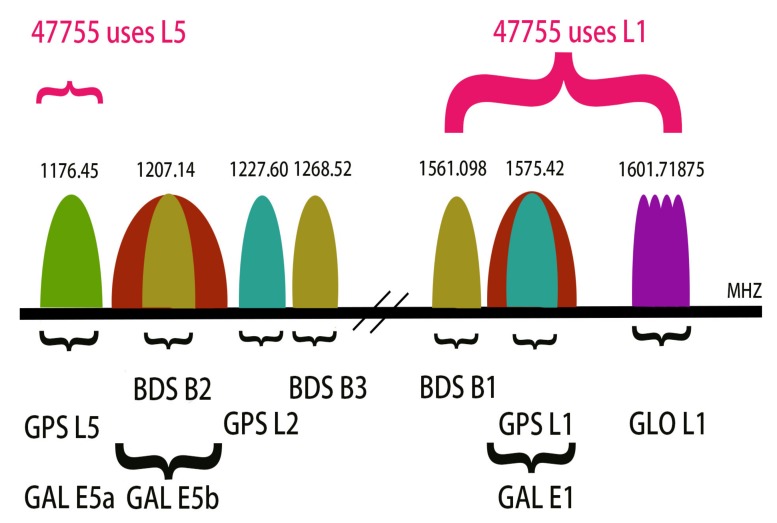
Visualization of the various GNSS carrier frequencies.

Our experiments were based on the Geo++ RINEX Logger and the Xiaomi Mi 8. The Xiaomi Mi 8 equipped with the BCM47755 could utilize dual-frequency signals, as Figure 2 shows.

All the Galileo satellites can broadcast E1 and E5a signals. In contrast, only 12 GPS satellites whose Block types are IIFcan broadcast the L5 signals [53]. A website called Trimble GNSS Planning can be helpful to identify a period when there are the most visible satellites that broadcast L1 (E1) and L5 (E5a) signals in a day [54]. We chose an M300 receiver made by ComNav Technology Ltd. Shanghai, China as the base station. This receiver can provide the measurements of all frequencies and all constellations. We also chose a dual-frequency NovAtel receiver, which supports L1 and L2 signals, as another rover. The position results provided by the dual-frequency RTK using the M300 receiver and the NovAtel receiver were regarded as the references.

#### 2.5.2. IMU Raw Measurements Collection

**Figure 3:**
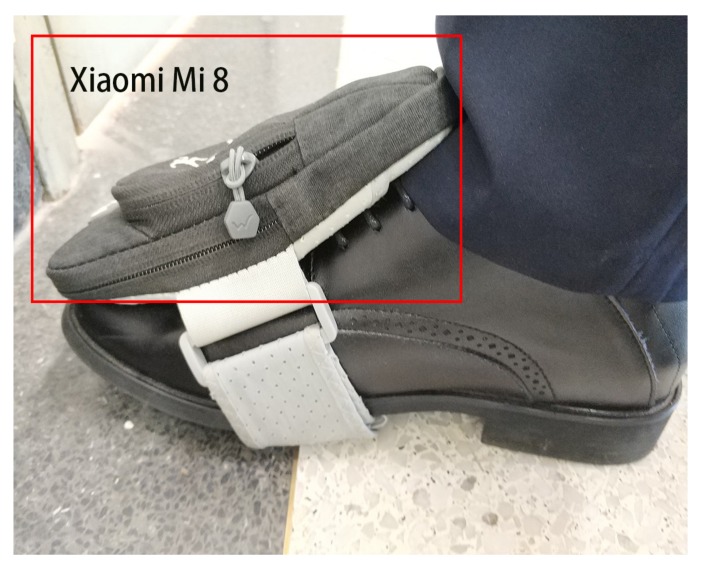
Close-up of tying the Xiaomi Mi 8 to our foot.

Here, we used an app called Sensorstream IMU+GPS to collect the measurements of accelerometers, gyroscopes, and magnetometers [55]. We tied the Xiaomi Mi 8 to our foot, as Figure 3 shows.

Sensorstream IMU+GPS can record the measurements of the sensors in smartphones and produce the measurement file in CSV format. Each measurement had its corresponding timestamp. The timestamp is the span from the booting to the happening of the sensor event. We wrote an app to get the time since the phone was booted to help transfer the timestamp of the sensor event to the UTC. There is also a difference between GPS time and UTC. We can transfer UTC to GPS time as Equation (44) shows up to 31 May 2019.

(44) $$t_{GPS}=t_{UTC}+18s$$

## 3. Results

### 3.1. RTK Performances Based on the Xiaomi Mi 8

#### 3.1.1. RTK Performances in Static Mode

**Figure 4:**
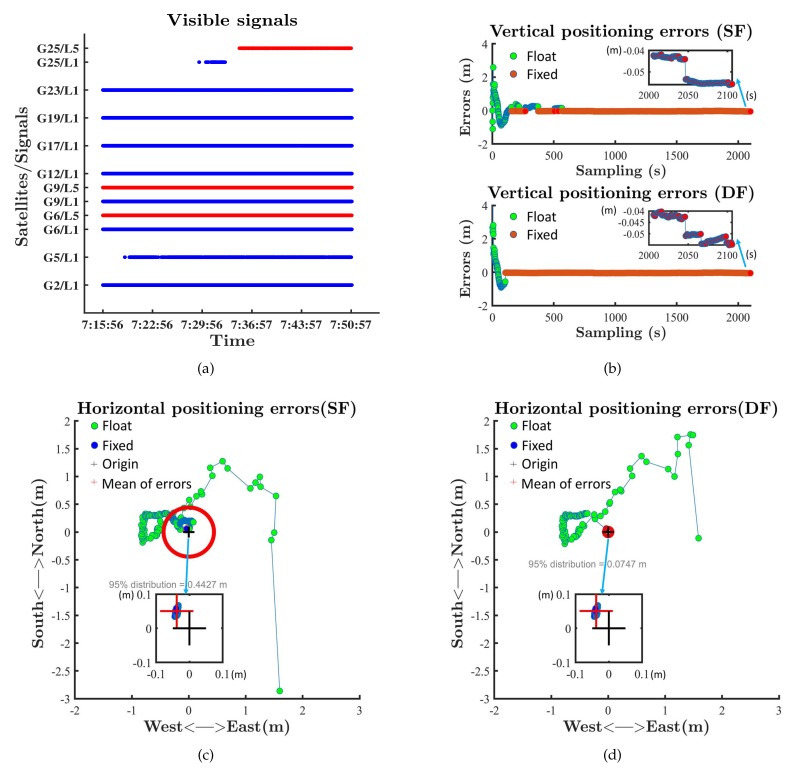
RTK performances in static mode. (**a**) The visible signals; (**b**) vertical positioning errors; (**c**) horizontal positioning errors (single frequency); (**d**) horizontal positioning errors (dual frequency).

We tested the RTK performance in static mode on the roof of the Yifu Building of Peking University on 12 July 2019. The baseline was 15 m. We tested the single-frequency RTK and dual-frequency RTK with GPS measurements. The visible signals are shown in Figure 4a. At least two L5 signals were visible during the test. Figure 4b–d show that both single-frequency RTK and dual-frequency RTK could provide fixed solutions and cm-level precision after a few minutes in static mode. The introduction of L5 measurements reduced the convergence time and improved the fixed rate.

#### 3.1.2. RTK Performances in Dynamic Mode

**Figure 5:**
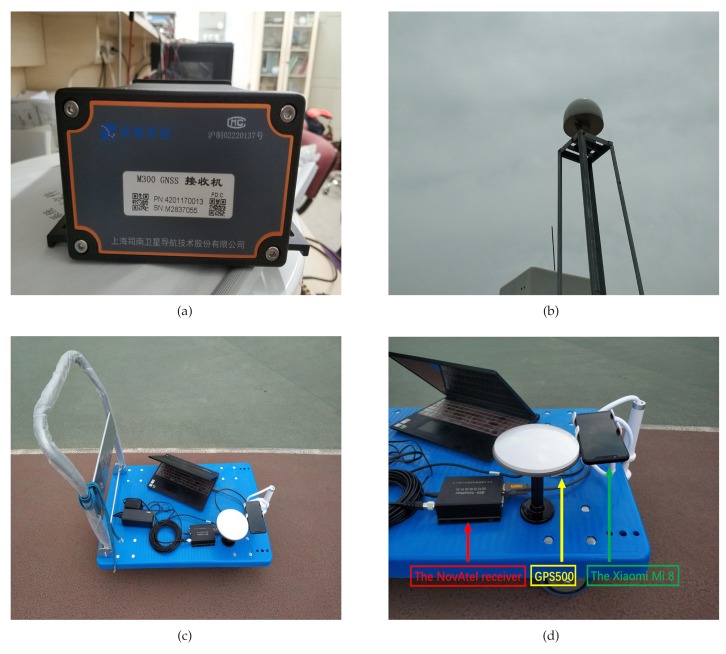
View of the base station and the rover. (**a**) View of the M300 receiver; (**b**) view of the antenna on the roof; (**c**) view of the rover; (**d**) brief description of the rover.

This section shows RTK performances in dynamic mode. We chose an M300 receiver as the base station (Figure 5a). The M300 receiver was capable of tracking all the signals of all the constellations. The M300 receiver was connected with an antenna located on the roof of our laboratory (Figure 5b). This antenna was also able to receive all the signals of all the constellations. We put the Xiaomi Mi 8 on a small handcart as the rover. We also located a receiver equipped with an OEM617-CDS-R0G-550 card close to the Xiaomi Mi 8 for comparison. This card that could track the L1/L2 signal of GPS was manufactured by NovAtel. The NovAtel receiver was connected to a mini-survey antenna GPS500, which was capable of receiving GPS L1/L2 (Figure 5c,d). The GPS500 was much more resistant to the influence of multipath and noise than the antenna in smartphones. We treated the dual-frequency RTK results provided by the NovAtel receiver as the reference.

**Figure 6:**
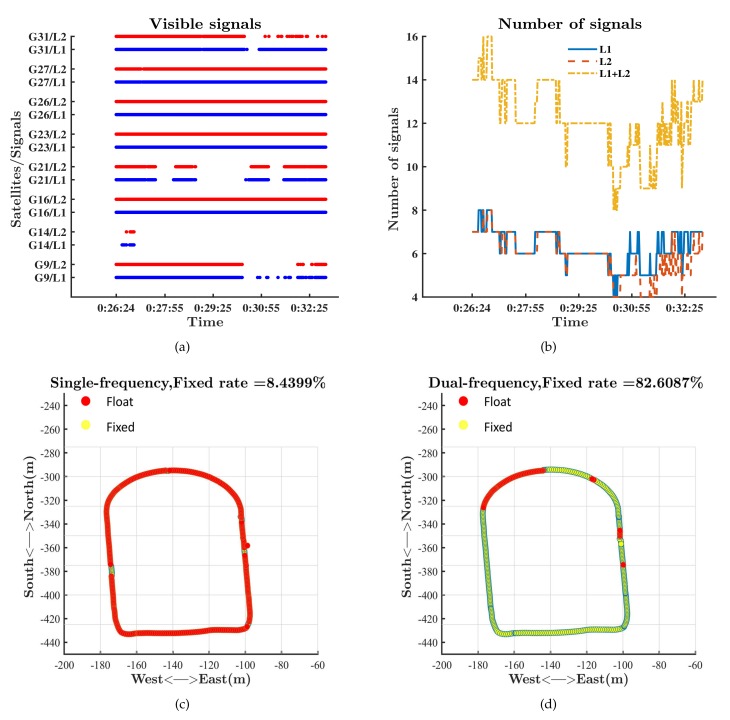
RTK performances based on the NovAtel receiver. (**a**) Visible signals and satellites; (**b**) number of different signals); (**c**) single-frequency RTK; (**d**) dual-frequency RTK.

We tested the RTK performance in dynamic mode on the sports ground of Peking University on 31 May 2019. We started from the north of the sports ground and walked counterclockwise. A tall building was located to the southeast of the sports ground, which could block signals. We compared the performance between single-frequency RTK and dual-frequency RTK with the NovAtel receiver. Signals tracked by the NovAtel receiver are shown in Figure 6a,b. All the signals shown in Figure 6a,b were tracked by both the rover and base station. The receiver could track the signals of at least four satellites thanks to the high quality of GPS500, which helped to keep providing positioning results during the test.

**Figure 7:**
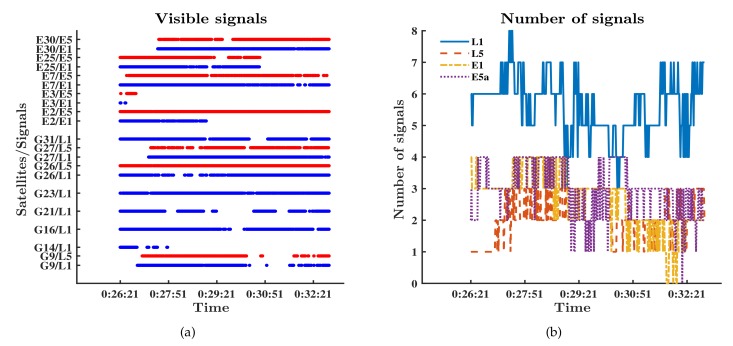
Signals tracked by the Xiaomi Mi 8. (**a**) Visible signals and satellites; (**b**) number of signals.

The performances of single-frequency RTK and dual-frequency RTK with the NovAtel receiver are presented in Figure 6c,d. The fixed rate of ambiguity resolution for single-frequency RTK was less than 9%, while that for dual-frequency RTK exceeded 82%. The differences between the results verified the conclusion that dual-frequency RTK was much more likely to get fixed ambiguity resolution than single-frequency RTK. We chose the dual-frequency RTK results as the reference to compare the performance of RTK in different modes based on the Xiaomi Mi 8. We divided RTK into four modes: RTK with L1 signals of GPS; RTK with L1 signals and L5 signals of GPS; RTK with L1 signals of GPS and E1 signals of Galileo; RTK with L1/L5 signals of GPS and E1/E5a signals of Galileo. Signals tracked by the Xiaomi Mi 8 are shown in Figure 7. Comparison of Figure 7 and Figure 6 explains that the Xiaomi Mi 8 had a little lower capacity of tracking GPS L1 signals than the NovAtel receiver because of the antenna with poor performance. As mentioned in the Introduction, the GPS constellation has more operational satellites than the Galileo constellation does. As a result, there are more GPS visible satellites at the same time. Figure 7 reflects that the number of Galileo satellites tracked by the Xiaomi Mi 8 was less than the number of GPS satellites tracked by the Xiaomi Mi 8. Figure 7 also reflects that the number of visible GPS satellites with both L1 and L5 signals was fewer than that of visible GPS satellites with only L1 signals. We can refer to the Trimble GNSS Planning to find the moments when there were enough visible GPS satellites that were broadcasting L5 signals and visible Galileo satellites.

**Figure 8:**
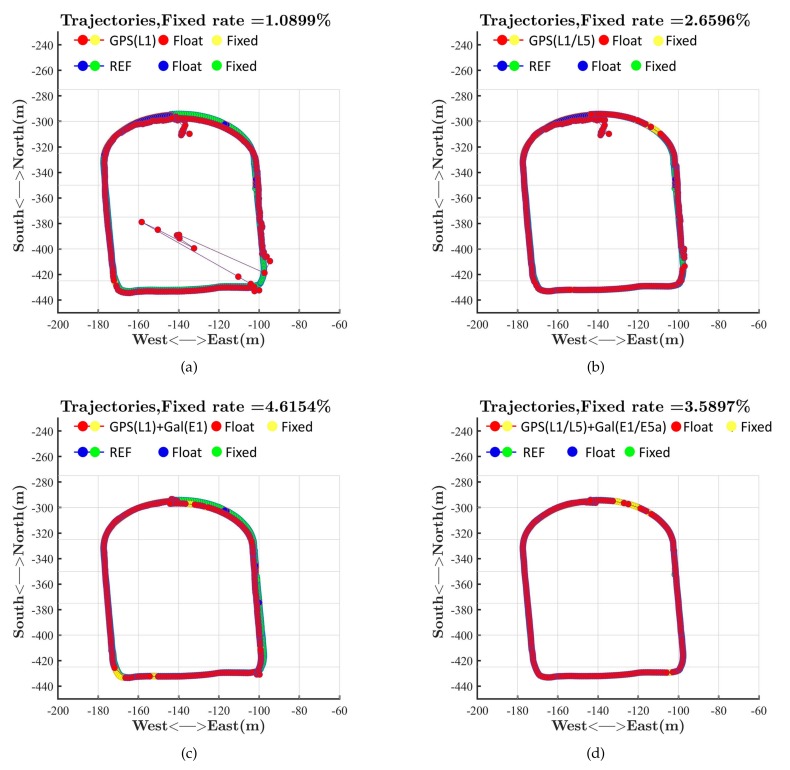
RTK performances on the sports ground. (**a**) GPS(L1); (**b**) GPS(L1 + L5); (**c**) GPS(L1) + Galileo(E1); (**d**) GPS(L1 + L5) + Galileo(E1 + E5a).

**Figure 9:**
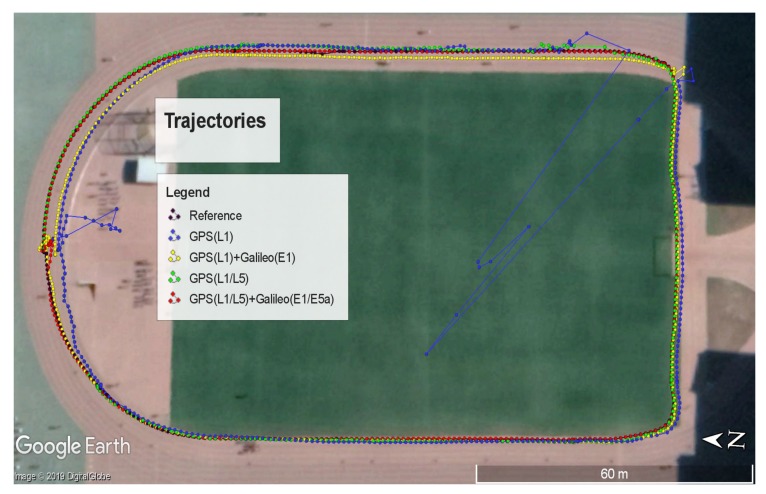
RTK performances shown in Google Earth.

The performances of RTK in different modes are presented in Figure 8 and Figure 9. Figure 8a shows the performance of RTK with only L1 measurements of GPS. The positioning results fluctuated drastically when the rover was located in the southeast of the sports ground because of the building nearby. This building blocked the signal and exacerbated the multipath effect, which could cause a big positioning error. RTKLIB ignored a satellite if the difference between its pseudorange and carrier phase exceeded the threshold, which was caused by the multipath effect and noise. If we only utilized the GPS, the number of valid satellites was likely to be less than four, which led to the absence of positioning results at some moments. As a result, Figure 8a shows that RTK with only L1 measurements did not provide positioning results in the southeast of the sports ground at some moments. Figure 8a also shows that the positioning results suffered from fluctuations and biases when the rover was located in the north of the sports ground at the beginning of the test, which reflected that RTK spent a few seconds converging to provide precise positioning results. Figure 8b shows the performance of RTK with L1 measurements and L5 measurements of GPS. The introduction of L5 measurements helped flatten the fluctuations caused by the blocking of signals and multipath effects. The positioning results given by RTK with L1 measurements and L5 measurements of GPS were also closer to the reference than those given by RTK with L1 measurements in Figure 8a, which meant more accuracy. However, the introduction of L5 signals could not increase the number of visible satellites. As a result, RTK still could not provide positioning results at some moments in the southeast of the sports ground. The introduction of L5 signals did not reduce the convergence time at the beginning, either. Figure 7 shows that there was only one valid L5 signal at the beginning of the test, which limited the advantages of dual-frequency RTK. Figure 8c shows the performance of RTK with L1 measurements of GPS and E1 measurements of Galileo. The introduction of another constellation meant more visible satellites. These additional Galileo satellites ensured the number of visible satellites when some GPS signals were blocked by the building, which helped to keep providing positioning results in the southeast of the sports ground. The introduction of Galileo also helped reduce the fluctuations and the convergence time at the beginning thanks to the improvement of the spatial distribution. Figure 8d shows the performance of RTK with L1/L5 measurements of GPS and E1/E5a measurements of Galileo. The positioning results given by RTK with L1/L5 measurements of GPS and E1/E5a measurements of Galileo were very close to the reference, as the introduction of Galileo helped increase the number of visible satellites, and dual frequency helped flatten the fluctuations and reduce the convergence time. However, Figure 8d shows that dual frequency could not help increase the fixed rate of ambiguity resolution for RTK on the Xiaomi Mi 8 in dynamic mode. This phenomenon was caused by the poor quality of signals because of the poor performance of the antenna. The statistics of positioning error in the ECEF coordinate are shown in Table 1. Table 1 also shows that dual-frequency RTK could provide positioning results with smaller biases and fluctuations.

**Figure 10:**
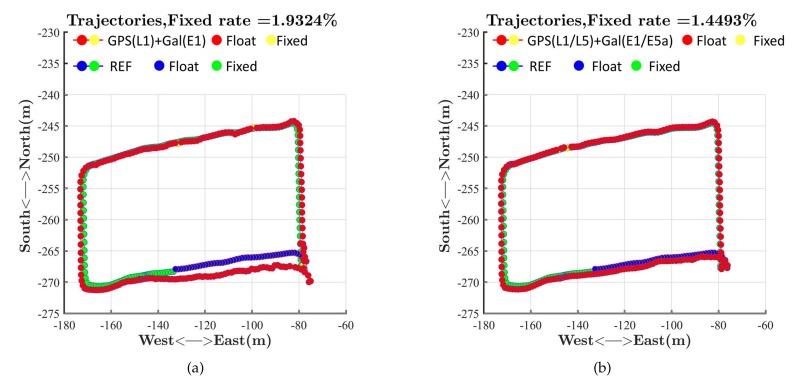
RTK performances on the basketball court. (**a**) GPS(L1) + Galileo(E1); (**b**) GPS(L1 + L5) + Galileo(E1 + E5a).

We tested the RTK performance in dynamic mode repeatedly to verify the advantages of dual-frequency RTK with the Xiaomi Mi 8. The results of a test on the basketball court are shown in Figure 10. The statistics of positioning errors in the ECEF coordinate are shown in Table 2.

Figure 10 and Table 2 also show that dual-frequency measurements could reduce the convergence time and positioning bias. Besides, Figure 10 shows that the fixed rate of ambiguity resolution for RTK on the Xiaomi Mi 8 in dynamic mode did not improve although the dual-frequency measurements were introduced.

### 3.2. The Performance of the Madgwick Algorithm

**Figure 11:**
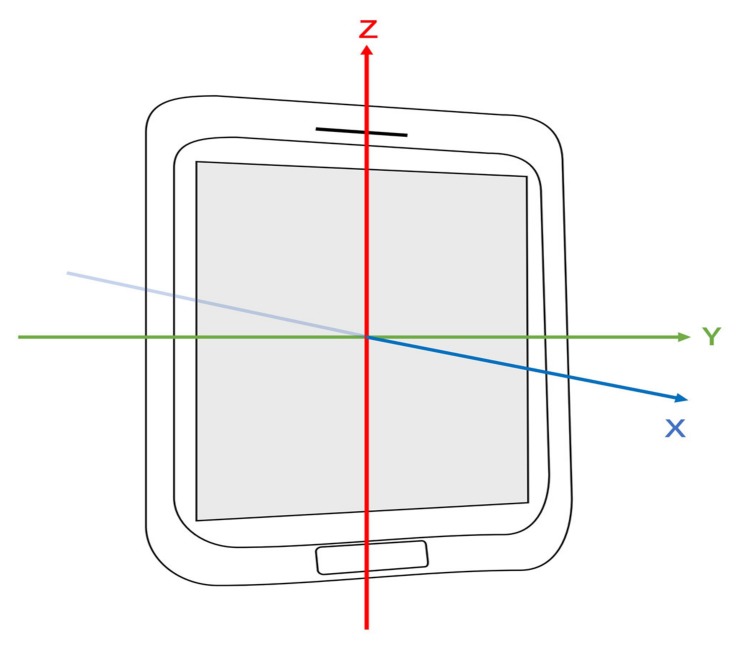
The coordinate system used by the Android system.

This section shows the performance of the Madgwick algorithm. We can use not only quaternion, but also Euler angles including pitch θ, roll γ, and yaw ψ to depict the attitude. Quaternion and Euler angles can be transformed into each other. Figure 11 shows the definition of the coordinate system used by the Android system [56]. The X-axis is horizontal and points to the right of the phone. The Y-axis is vertical and points to the top of the phone. The X-axis and Y-axis are in a plane parallel to the screen of the phone’s surface. The Z-axis points towards the outside of the front face of the screen and is perpendicular to the plane of the X-axis and Y-axis. Yaw is defined as the angle between the magnetic north direction and the Y-axis, which reflects the rotation around the Z-axis. Yaw values range from $0^{\circ }$–$360^{\circ }$ where $0^{\circ }=$ north, $90^{\circ }=$ east, $180^{\circ }=$ south and $270^{\circ }=$ west. Pitch reflects the rotation around the X-axis with positive values when the Y-axis moves toward the Z-axis. Pitch values range from $-90^{\circ }$–$+90^{\circ }$ where $0^{\circ }=$ horizon, $+90^{\circ }=$ straight up, and $-90^{\circ }=$ straight down. Roll reflects the rotation around the Y-axis with positive values when the X-axis moves toward the Z-axis. Roll values range from $-180^{\circ }$–$+180^{\circ }$.

**Figure 12:**
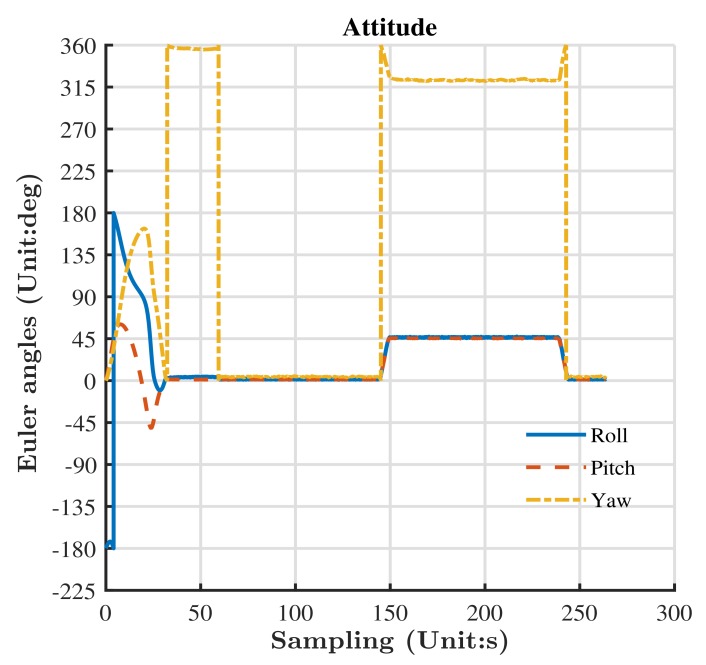
The performance of the Madgwick algorithm.

We changed the Xiaomi Mi 8’s attitude to test the performance of the Madgwick algorithm with a three-axis turntable. The turntable could provide an accurate attitude with an error of smaller than four seconds. We initiated the turntable, setting the pitch, roll, and yaw to zero. Then, we changed the pitch, roll, and yaw of the turntable simultaneously. We set the pitch and roll to $45^{\circ }$, while we set yaw to $325^{\circ }$. After that, the three Euler angles were set to zero again. Since the Xiaomi Mi 8 was fixed in the chute of the turntable, the attitude of the Xiaomi Mi 8 was strictly in line with the attitude of the turntable. We collected the measurements of sensors and estimated the Euler angles with the Madgwick algorithm. The results are shown in Figure 12. The estimated Euler angles corresponded to the change of attitude.

### 3.3. The Performance of the ZUPT Aiding INS

**Figure 13:**
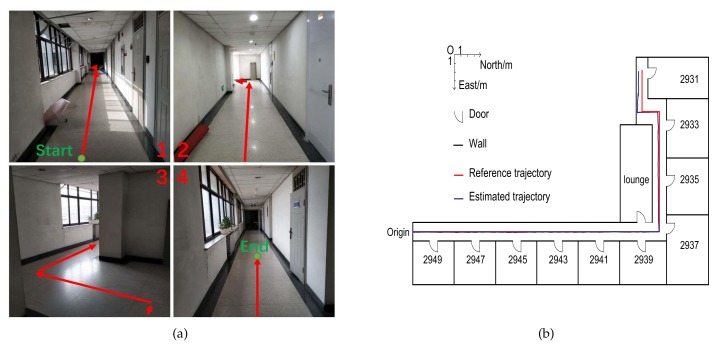
The performance of the ZUPT aiding INS. (**a**) Place where we walked; (**b**) the estimated trajectory.

This section shows the performance of the ZUPT aiding INS. We tied the Xiaomi Mi 8 to our foot and took a walk following the pre-planned path (approximately 50 meters) in the corridor on the ninth floor of the Yifu Building of Peking University. The place where we walked is shown in Figure 13a, and the pre-planned path is shown by the red arrows in Figure 13a. We measured the length and width of the corridor and established a model of the environment with AutoCAD. The results of the ZUPT aiding INS are shown in this model in Figure 13b. It can be seen that the results could reflect our trajectory.

### 3.4. Performances of RTK with the Assistance of an IMU-Based Pedestrian Navigation Algorithm

**Figure 14:**
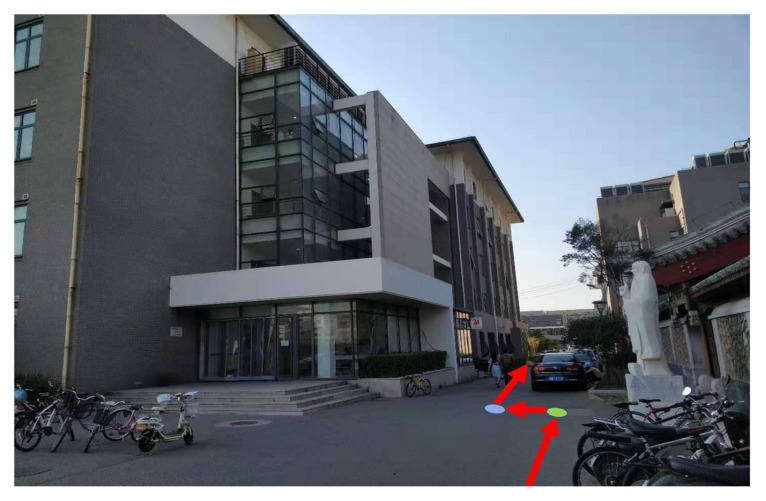
A narrow path with tall teaching buildings on both sides.

This section shows the performance of RTK with the assistance of an IMU-based pedestrian navigation algorithm. We walked through a narrow path with tall teaching buildings on both sides, as Figure 14 shows. We walked following the red arrows and turned left at the green point. We turned right at the blue point with a five-meter distance between the two points. We kept going straight at other moments.

**Figure 15:**
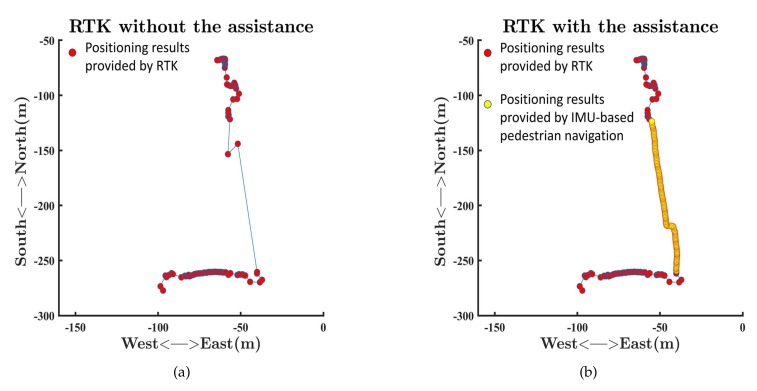
Comparison between the performances of RTK without the assistance of an IMU-based pedestrian navigation algorithm and RTK with the assistance of an IMU-based pedestrian navigation algorithm. (**a**) The performance of RTK without the assistance of an IMU-based pedestrian navigation algorithm; (**b**) the performance of RTK with the assistance of an IMU-based pedestrian navigation algorithm.

**Figure 16:**
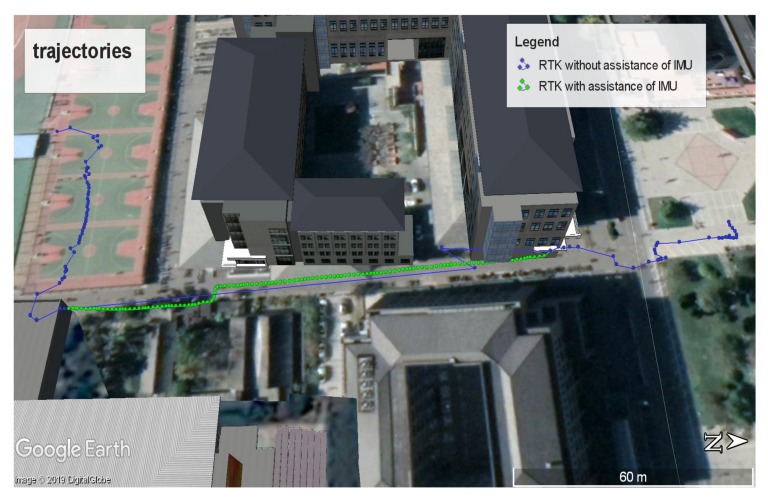
Trajectories in Google Earth.

Tall buildings could not only block GNSS signals, but also aggravate the multipath effect, which hindered RTK from providing positioning results, as Figure 15a shows. There were only two points between the two blue points in Figure 15a, which meant RTK could hardly provide the positioning results because of the GNSS outage. Although the RTK could provide two positioning results as the green point and the black point shown in Figure 15a, the positioning results were not precise enough. The black point was to the southwest of the green point, which was incompatible with the fact that we kept walking toward roughly north. Severe multipath effects in the urban canyon limited the accuracy of RTK. As a result, it was unreliable to provide positioning results with RTK based on the Xiaomi Mi 8 in the urban canyon. We tied the Xiaomi Mi 8 to our foot to utilize the ZUPT aiding INS to provide continuous positioning results during GNSS outage, as mentioned in the Materials and Methods. The positioning results are shown in Figure 15b. The outputs of the ZUPT aiding INS were able to fill in the position when the positioning results of the RTK were lost, as Figure 15b shows. Positioning results in Figure 15b could reflect our trajectory as there were two changes in the direction of motion. The last output of the ZUPT aiding INS was consistent with the first positioning result provided by the RTK after we passed through the urban canyon. Figure 15 and Figure 16 show that the combination of RTK, the Madgwick algorithm, and the ZUPT aiding INS could be more robust and more reliable than RTK without the assistance of an IMU-based pedestrian navigation algorithm. RTK with the assistance of an IMU-based pedestrian navigation algorithm could be more adaptable to urban areas because of the capacity of providing positioning results during GNSS outage. Our experiment demonstrates the feasibility of providing continuous positioning results by using RTK with the assistance of an IMU-based pedestrian navigation algorithm.

## 4. Conclusions and Future Work

In this paper, we tested the performances of single-frequency RTK and dual-frequency RTK on the Xiaomi Mi 8 and compared the performance of RTK based on the Xiaomi Mi 8 with that based on the NovAtel receiver. Both single-frequency RTK and dual-frequency RTK could provide fixed solutions in static mode. The introduction of the dual-frequency RTK reduced the convergence time and improved the fixed rate in static mode. When it comes to dynamic mode, dual-frequency RTK with GPS and Galileo measurements suffered from the least biases and fluctuations. The introduction of Galileo satellites helped provide more continuous positioning results because of the increasing number of visible satellites. The fixed rate of dual-frequency RTK based on the Xiaomi Mi 8 in dynamic mode did not rise significantly compared with single-frequency RTK based on the Xiaomi Mi 8.

We also provided a method to assist RTK with an IMU-based pedestrian navigation algorithm for smartphones. We combined RTK with the Madgwick algorithm and the ZUPT aiding INS to provide more continuous positioning results. The experiment showed that RTK with the assistance of IMU could provide reliable positioning results during GNSS outage, which was much more reliable and adaptable to complex environments than RTK without the assistance of an IMU-based pedestrian navigation algorithm. The experiments verified the feasibility of providing continuous positioning results in both GNSS-allowed and GNSS-challenged environments with a Xiaomi Mi 8.

Assisting RTK with an IMU-based pedestrian navigation algorithm is still in the proof-of-concept stage. Experiments verified the feasibility of our method. However, further work is needed to assess our method’s performance more accurately. In the future, we will conduct experiments to test the method’s accuracy and stability in different environments. Besides, we will obtain the GNSS measurements and measurements of IMU with our app and develop a GNSS/INS with a loosely-coupled or tightly-coupled integration system based on smartphones to raise the anti-jamming capability of RTK. Considering tying smartphones to the pedestrian’s foot is inconvenient and not practical for pedestrians, we will also assist RTK with other algorithms during GNSS outage so that continuous positioning results can be provided with a handheld smartphone.

## Figures and Tables

**Table 1:** Statistics of positioning errors in the ECEFcoordinate (sports ground).

Modes		GPS(L1)	GPS(L1/L5)	GPS(L1) + Gal(E1)	GPS(L1/L5) + Gal(E1/E5a)
	X	2.4395	1.3852	0.3424	0.0111
Mean (*m*)	Y	−5.2601	−1.5211	−1.5423	0.2136
	Z	−2.2977	−1.1398	−0.0815	0.1150
	Norm	6.2370	2.3520	1.5819	0.2428
	X	7.8644	3.3493	1.2287	0.5905
RMS (*m*)	Y	5.8325	3.7130	3.0119	0.9283
	Z	5.3058	1.9419	0.8935	0.4540
	Norm	11.1364	5.3643	3.3734	1.1902

**Table 2:** Statistics of positioning errors in the ECEF coordinate (basketball court).

Modes		GPS(L1) + Gal(E1)	GPS(L1/L5) + Gal(E1/E5a)
	X	0.2175	0.2002
Mean (*m*)	Y	−1.6638	−1.1142
	Z	−0.4090	−0.5422
	Norm	1.7271	1.2552
	X	0.7182	0.3727
RMS (*m*)	Y	1.8605	1.4237
	Z	1.9676	1.2460
	Norm	2.8015	1.9284
